# Relationship between ABO blood group and Acute Lymphoblastic Leukemia

**Published:** 2014-03-15

**Authors:** F Tavasolian, E Abdollahi, M Vakili, A Amini

**Affiliations:** 1 Department of Immunology, Faculty of Medicine, Shahid Sadoughi University of Medical Sciences, Yazd, Iran; 2 Department of Community Medicine, Faculty of Medicine, Shahid Sadoughi University of Medical Sciences, Yazd, Iran; 3 School of Paramedical Sciences,Shahid Sadoughi University of Medical Sciences,Yazd, Iran.

**Keywords:** Acute lymphoblastic leukemia, ABO blood group, Children

## Abstract

Acute lymphoblastic leukemia (ALL) constitute a family of genetically heterogeneous lymphoid neoplasms derived from B- and T-lymphoid progenitors. ALL affects both children and adults. Diagnosis is based on morphologic, immunophenotypic, and genetic features that allow differentiation from normal progenitors and other hematopoietic and nonhematopoietic neoplasms. The aim of this study was to investigate the association between ALL and ABO blood group.

**Material and method:**

This is a case-control study that was carried out in Amir Oncology Hospital in Shiraz during 2011 to2013. The case group consisted of 293 patients with acute lymphoblastic leukemia. And compared with 300 subject in control group ( the age in the case group was between 2-5 year, and the age in the control group was between 2-45 year) .Statistical analyzes was done performed by chi –square test.

The results was considered significant when p value <0.05. (CI:0.95)

**Results:**

The ABO blood group distribution was 82(A), 59 (B), 24 (AB) and 128(O) in patient with Acute Lymphoblastic Leukemia and the blood group of 300 participants in the control group include, 63% (25) A, 69% (25.6) B, 18 % 06.8) AB and 101% (42.6) O. The ABO blood group distribution showed that there is significant differences between ABO blood group and patients with acute lymphoblastic leukemia .

**Conclusion:**

This study showed significant association between ALL and ABO blood group and showed that blood group AB was associated with a higher risk of All (p value<0.001).

## Introduction

Acute lymphoblastic leukemia (ALL) is a form of leukemia, or cancer of the white blood cells characterized by excess lymphoblasts. Malignant, immature white blood cells continuously multiply and are overproduced in the bone marrow. ALL causes damage and death by crowding out normal cells in the bone marrow, and by spreading to other organs. ALL is most common in childhood with a peak incidence at -2-5 years of age, and another peak in old age(1, 2) After the discovery of an association between stomach cancer and blood type A in 1953(3), there have been several studies on possible relationship of blood types to certain diseases. Using classical serological studies, it is possible to classify individuals into four blood groups (A, B, O, and AB)(4, 5). There are two antigens and two antibodies that are mostly responsible for the ABO types. The specific combination of these four components determines an individual's type in most cases (5). If the risk of Several different diseases are known for different ABO blood groups, it could serve as an epidemiological marker or a primary screening aid to identify high-risk populations(6).Hence, the distribution of ABO blood groups among patients with acute lymphoblastic leukaemia (ALL) was studied in this study.

## Materials and Methods

This is an un matched case-control study. This study was carried out in Amir Oncology Hospital in Shiraz .Sampling was performed during 2011-2013 . The case group consisted of 293 patients who was diagnosed with acute lymphoblastic leukemia by bone marrow aspiration. The blood group data were collected from the case records of patients with ALL. Determination of blood type was performed for the purpose of blood transfusion in the most number of the cases. Blood typing included confirmatory back-typing with patient serum. The control group consisted of 300 healthy participants that refered to the Amir Oncology Hospital . 

**Statistical Analysis**

Statistical analyzes was performed by chi –square test.The results was considered significant when p value <0.05 (CI:0.95).

## Results

109 participants of 293 patients with ALL were female and 184 were male. The ABO blood group distribution was 82(28%A), 59 (20.1%B), 24 (8.2%AB) and 128(43.7%O) in patient with ALL .The distribution of blood group of ALL patients was not differed significantly in male and female(p value=0.2).The ABO blood group distribution in control participants was 75(25%A), 82 (25.6%B), 22 (6.8%AB) and 121(45.6%O) . It has been observed that distribution of the ABO blood group in ALL patients was differed significantly compared to the control group . And there is a significant relationship between patients with ALL and group AB( P value <0.001).

**Table I:** Frequency of ABO blood types in ALL patient

**Blood group**	** SEX**				**Total**		**95% Confidence interval**	**P value**
	**male**		**female**					
	**n**	**%**	**n**	**%**	**n**	**%**		
**O**	**83**	**45.1**	**45**	**41.3**	**128**	**43.7**	**0.72-1.88**	**0.52**
**A**	**49**	**26.6**	**33**	**30.3**	**82**	**28.0**	**0.70-2.01**	**0.50**
**B**	**41**	**22.3**	**18**	**16.5**	**59**	**20.1**	**0.37-1.27**	**0.23**
**AB**	**11**	**6.0**	**13**	**11.9**	**24**	**8.2**	**0.91-4.93**	**0.07**
**Total**	**184**	**100.0**	**109**	**100.0**	**293**	**100.0**		

**Table II:** Distribution of ABO blood group in two groups

**P.value**	**95%CI**	**OR**	**%**	**Patient** **N**	** % **	**Control** ** N **	
**0.430**	**23-33**	**1.19**	**28**	**82**	**25**	**75**	**A**
**0.126**	**15.5-24.7**	**0.69**	**20.1**	**59**	**25.6**	**82**	**B**
**0.001**	**7.8-8.6**	**2.13**	**8.2**	**24**	**6.8**	**22**	**AB**
**0.796**	**38-49.4**	**1.16**	**43.7**	**128**	**45.6**	**121**	**O**
			**100**	**293**	**100**	**300**	

This table show that there is a significant difference between ABO blood group of patient with ALL and control group , and there is a significant relationship between patient with ALL and blood group AB ( P value :0.001).

**Figure 1:**
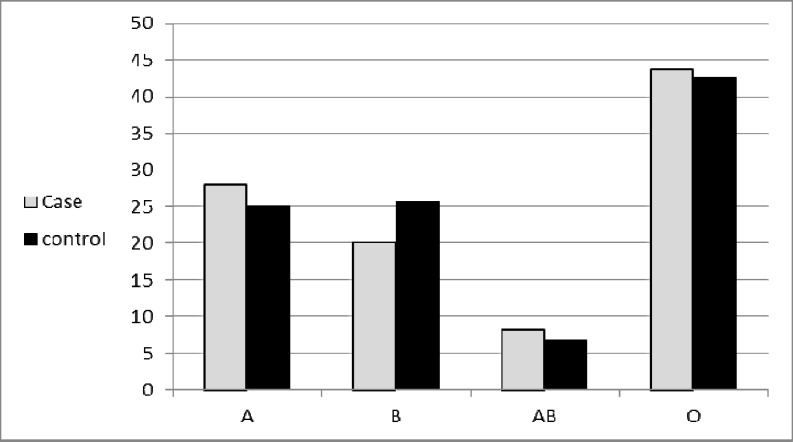
Distribution of ABO blood group in two groups

## Discussion

Acute lymphoblastic leukemia(ALL) is a clonal disease arising from somatic mutations in a lymphoid progenitor cell that alter regulation of cellular proliferation ,diffrentiation and apoptosis(7, 8). Rapid accumulation of precursor of lymphoid cells usually in the bone marrow displaced normal hematopoiesis resulting in neutropenia, thorombocytopenia, anemia, and dissemination of leukemic cells in the peripheral blood(8, 9). The antigens of the ABO system were the first to be recognized as blood groups and actually the first human genetic markers known(10, 11). Many studies have been published inconsistent results on the distribution of blood types in different disease(12). This study was performed to find any association of ABO blood groups in ALL patients. The results of the present study showed that there is a significant differences between ABO blood group and patients with acute lymphoblastic leukemia. This study showed that higher percentage of patients with AB blood type had ALL (P.value<0.001) .Various studies have reported conflicting results on the distribution of blood groups among acute leukaemias . Some of the studies discovered significant difference and higher percentage of O blood type among patients with acute leukemia(13).but other researchers have reported different results in their study(14). Nagy and colleagues showed an increase in the proportion of O blood group among female patients with acute leukaemia(15). Simona Iodis reported a significant difference in the distribution of O blood group in Hodgkin lymphoma and non-Hodgkin Lymphoma(16). Steinberg found no differenc in the distribution of ABO blood group among patients with acute leukaemias compared to the general population (17). Shirley and Desai reviewed several previously published data and found no statistically significant difference in the distribution of ABO blood group in patients with acute leukaemia when compared with the respective controls of each study reviewed(18) . MacMahon and Forman, found no relationship between ABO blood group and the length of survival of patients with the acute leukemias(19). For the chronic leukemias, Feinleib and MacMahon, noted that length of survival is significantly longer for patients of group B than for patients of other groups(20). Jackson and colleagues reported a decrease in the proportion of O blood group among female patients with acute leukaemias (21) . The result showed that there is a significant difference in the distribution of AB blood group in ALL patients compared to the control group . conflicting results or statistically not significant results of previous studies could have been due to AML being included with ALL as a single disease group . this study demonstrates an association between ABO blood groups and specific haematological malignancies, but because our data was collected in a single hospital , further reserche in a large population-based prospective study is need. 

## Conclusion

The present study revealed that there are significant differences between ABO blood group and patients with acute lymphoblastic leukemia. These findings also raise the possibility of using blood groups as an epidemiological marker for identifying population subgroups which are at high risk for these malignancies.
